# Supplementary material for: Simulating nationwide coupled disease and fear spread in an agent-based model
Source: Sci Rep. 2025 Nov 26;15:42235. doi: 10.1038/s41598-025-26425-y (PMC12658175; doi:10.1038/s41598-025-26425-y)
Supplement: Supplementary file 1 — Supplementary Information. [file 41598_2025_26425_MOESM1_ESM.pdf]

Supplementary material for: “Simulating nationwide coupled disease and fear spread in an agent based model”

Joy Kitson[1,2,*], Prescott C. Alexander[2,3], Joseph Tuccillo[4], Abhinav Bhatele[1], Sara Y. Del Valle[3], and Timothy C. Germann[2]

[1]Department of Computer Science, University of Maryland, College Park, 20740, USA
[2]Theoretical Division, Los Alamos National Laboratory, Los Alamos, 87544, USA
[3]Analytics, Intelligence and Technology Division, Los Alamos National Laboratory, Los Alamos, 87544, USA
[4]Geospatial Science and Human Security Division, Oak Ridge National Laboratory, Oak Ridge, 3730, USA
[*]jkitson@umd.edu

ABSTRACT

This document contains supplementary material to support the publication of the paper “Simulating nationwide coupled disease and fear spread in an agent based model”. This includes three main components. First is a specification of the full system of ordinary differential equations (ODEs) for the $SEPI_sI_aR_sR_a \times NF$ model, which incorporates all disease states used by the EpiCast agent-based model (ABM). The second is a specification of the values of key parameters used in the experiments conducted in the main paper. The third is an additional set of sensitivity analysis experiments designed to investigate the impact of varying initial conditions, random seeds, and easily broadcasters begin spreading fear.

S1 Coupled contagions ODE models

We provide the full formulation of the system of ODEs which define the $SEPI_sI_aR_sR_a \times NF$ model below. This system is summarized in the flow diagram in Supplementary Fig. S1. Let $<x,y>$ be the size of the compartment with disease state x and fear state y. The $SI_sI_aR_sR_a \times NF$ model can be obtained by removing all terms involving the **E**xposed and **P**resymptomatic infectious disease states (i.e. $<E,N>, <E,F>, <P,N>$, and $<P,F>$). The $SIR \times NF$ model may similarly be obtained by additionally removing all terms involving **a**symptomatic disease states (i.e. $<I_a,N>, <I_a,F>, <R_a,N>$, and $<R_a,F>$). The system of ODEs is as follows:

fear transmission rate

$$\text{fear}_\uparrow(<\bullet,N>) = \beta_f \left(<I_s,N> + \sum_x <x,F>\right) <\bullet,N>$$

$$\text{fear}_\downarrow(<\bullet,F>) = \left(\gamma_f + \alpha_f \cdot <R_s,N>\right) <\bullet,F>$$

baseline fear loss rate — fear loss rate from contact with symptomatic recovered

disease transmission rate — relative infectivity of fearful individuals

$$\begin{aligned}\text{disease}_\uparrow(<S,\bullet>) = \beta\,(&<I_s,N> + \iota_f \cdot <I_s,F> \\ &+ \iota_a\,(<P,N> + <I_a,N> \\ &+ \iota_f\,(<P,F> + <I_a,F>)))<S,\bullet>\end{aligned}$$

relative infectivity of asymptomatic individuals

disease recovery rate

$$\text{disease}_\downarrow(<x,\bullet>) = \gamma \cdot <x,\bullet> : x \in \{I_s, I_a\}$$

$$\begin{aligned}\frac{d<S,N>}{dt} = &-\text{disease}_\uparrow(<S,N>) \\ &-\text{fear}_\uparrow(<S,N>) + \text{fear}_\downarrow(<S,F>)\end{aligned}$$

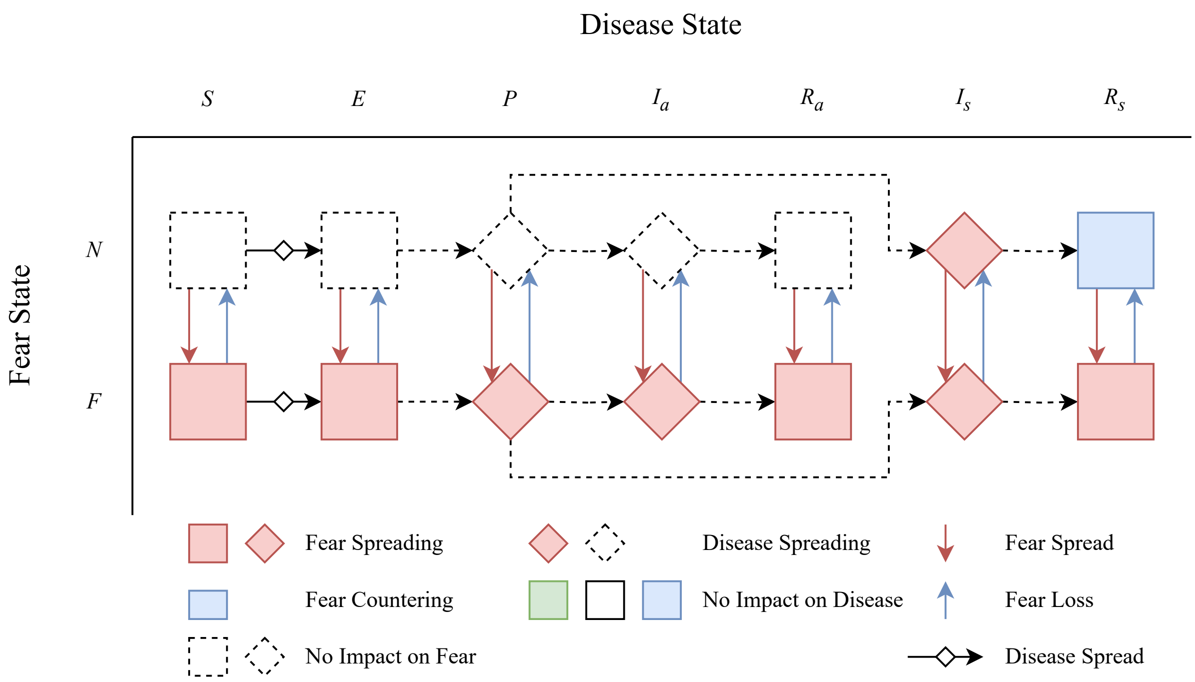

Supplementary Figure S1. **Flow diagram for $SEPI_sI_aR_sR_a \times NF$ coupled contagion ODE model**

disease susceptibility reduction from fear

$$\frac{d<S,F>}{dt} = -\sigma_f \cdot \text{disease}_{\uparrow}(<S,F>) + \text{fear}_{\uparrow}(<S,N>) - \text{fear}_{\downarrow}(<S,F>) \tag{12}$$

inverse of incubation period

$$\begin{aligned} \frac{d<E,N>}{dt} &= \text{disease}_{\uparrow}(<S,N>) - \delta \cdot <E,N> + \text{fear}_{\uparrow}(<E,N>) + \text{fear}_{\downarrow}(<E,F>) \\ \frac{d<E,F>}{dt} &= \sigma_f \cdot \text{disease}_{\uparrow}(<S,F>) + \delta \cdot <E,N> - \text{fear}_{\uparrow}(<E,N>) + \text{fear}_{\downarrow}(<E,F>) \\ \frac{d<P,N>}{dt} &= \delta \cdot <E,N> - <P,N> \\ \frac{d<P,F>}{dt} &= \delta \cdot <E,F> - <P,F> \end{aligned} \tag{13}$$

probability of symptomatic infection

$$\begin{aligned} \frac{d<I_s,N>}{dt} &= p_s \cdot <P,N> - \text{disease}_{\downarrow}(<I_s,N>) - \text{fear}_{\uparrow}(<I_s,N>) + \text{fear}_{\uparrow}(<I_s,F>) \\ \frac{d<I_s,F>}{dt} &= p_s \cdot <P,N> - \text{disease}_{\downarrow}(<I_s,N>) + \text{fear}_{\uparrow}(<I_s,N>) - \text{fear}_{\uparrow}(<I_s,F>) \end{aligned} \tag{14}$$

probability of asymptomatic infection

$$\frac{d < I_a, N >}{dt} = p_a \cdot < P, N > - \text{disease}_{\downarrow}(< I_a, N >)$$
$$- \text{fear}_{\uparrow}(< I_a, N >) + \text{fear}_{\uparrow}(< I_a, F >)$$
$$\frac{d < I_a, F >}{dt} = p_a \cdot < P, F > - \text{disease}_{\downarrow}(< I_a, N >)$$
$$+ \text{fear}_{\uparrow}(< I_a, N >) - \text{fear}_{\uparrow}(< I_a, F >)$$

$$\frac{d < R_s, N >}{dt} = \text{disease}_{\downarrow}(< I_s, N >)$$
$$- \rho_f \cdot \text{fear}_{\uparrow}(< R_s, N >) + \text{fear}_{\uparrow}(< R_s, F >)$$
$$\frac{d < R_s, F >}{dt} = \text{disease}_{\downarrow}(< I_s, F >)$$
$$+ \rho_f \cdot \text{fear}_{\uparrow}(< R_s, F >) - \text{fear}_{\uparrow}(< R_s, F >)$$

fear susceptibility reduction from symptomatic recovery

$$\frac{d < R_a, N >}{dt} = \text{disease}_{\downarrow}(< I_a, N >)$$
$$- \text{fear}_{\uparrow}(< R_a, N >) + \text{fear}_{\uparrow}(< R_a, F >)$$
$$\frac{d < R_a, F >}{dt} = \text{disease}_{\downarrow}(< I_a, F >)$$
$$+ \text{fear}_{\uparrow}(< R_a, F >) - \text{fear}_{\uparrow}(< R_a, F >)$$

S2 Model parameters

Parameter values used in ODE model comparison experiments (Section 4.1) are shown in Supplementary Table S1 while those used in the EpiCast scenario comparison experiments (Section 4.2) are shown in Supplementary Table S2. Where possible, sources for the values used are provided. For rows in which multiple values are used, the values without a citation are generally chosen to contrast with the cited values.

S3 Additional Sensitivity Analysis

We conduct another set of experiments which vary the distribution of initial cases used to seed infections in EpiCast, along with the values of $p_{\text{bc\_start}}$, using several different random seeds for each cell. For these runs, we use the population of the U.S. state of Colorado ($\sim$ 5.6 million agents) with three different starting conditions:

1. March 25: Infections and immune agents are seeded with case data from March 25 2020, similar to the other EpiCast runs presented above.
2. Denver only: Infections and immune agents are seeded only in Denver County.
3. All counties: Infections and immune agents are seeded in equal number in every county of Colorado. Counts are not scaled by the population of the county.

Note that in all cases we seed approximately the same number of infections and immune agents, though in the final case we round down the per-county averages. In all cases we use the parameter values from Scenario (f) except when otherwise specified.

We run two sets of simulations. In the first, shown in Fig. S2, we use $\sigma_f = 0$ and $p_{\text{fear}} = 0.25$ to test a set of parameters that produced a single wave for the state of Colorado experiments. In the second, shown in Fig. S3 we use $\sigma_f = 0.5$ and $p_{\text{fear}} = 0$ to test a set of parameters that produces two waves.

For the first set of runs, we observe relatively little variation based on random seed within a given cell. We also note that larger values of $p_{\text{bc\_start}}$ as much as doubles the peak rate of new infections. With respect to initial conditions, seeding based on the observed cases from March 25 produces the most infections, given the same value of $p_{\text{bc\_start}}$. All cases produce one wave.

For the second set of runs all cases produce two waves. Here we observe greater variability for $p_{\text{bc\_start}} = 0.75$, along with much greater decay in fear levels from their initial peak and a corresponding heightened second peak of the epidemic. Differences in cases due to initial conditions are relatively minor in this regime.

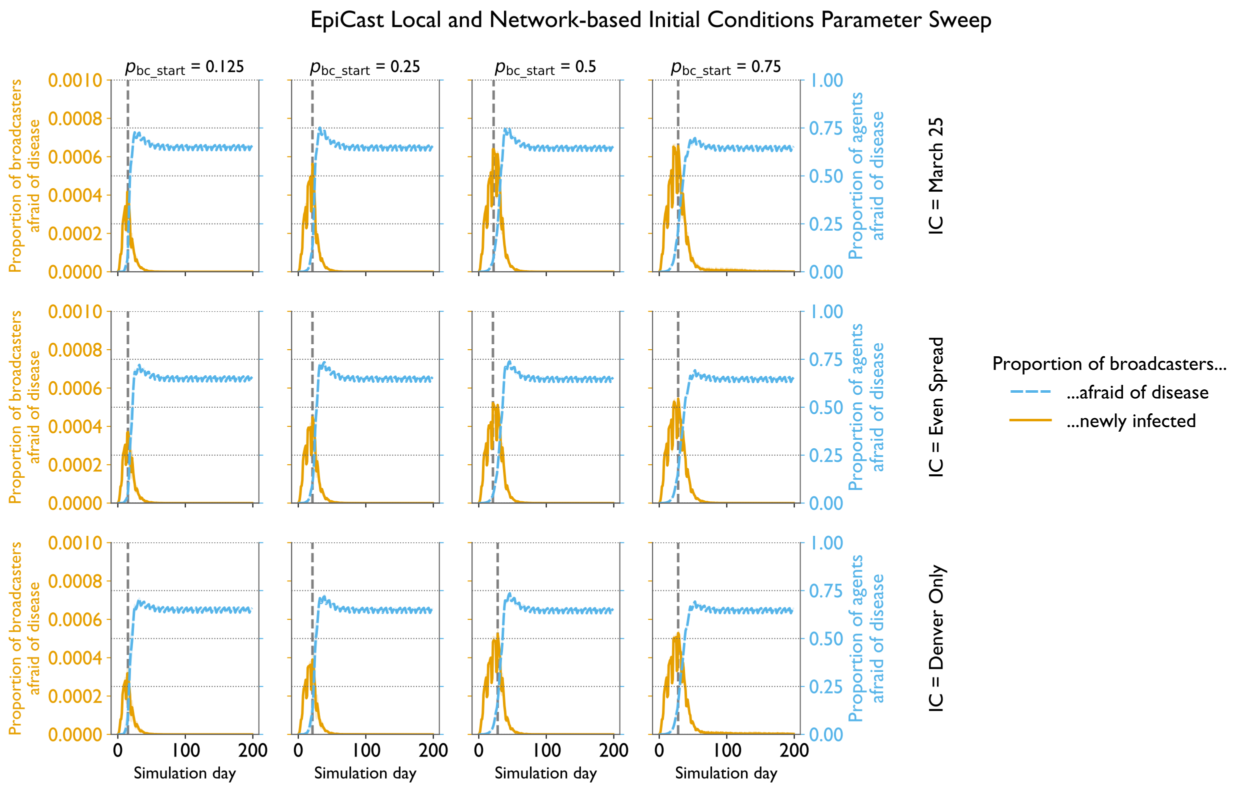

Supplementary Figure S2. **Epicast one-wave initial condition and broadcaster threshold sensitivity analysis:** New cases by day (with peaks indicated by dashed vertical line), for various initial conditions (IC) and thresholds for broadcasters to start spreading fear (p_{bc_start}) with both local and broadcaster-based fear spread. All lines represent the average of three replicates, with a 95% confidence interval shown in shading.

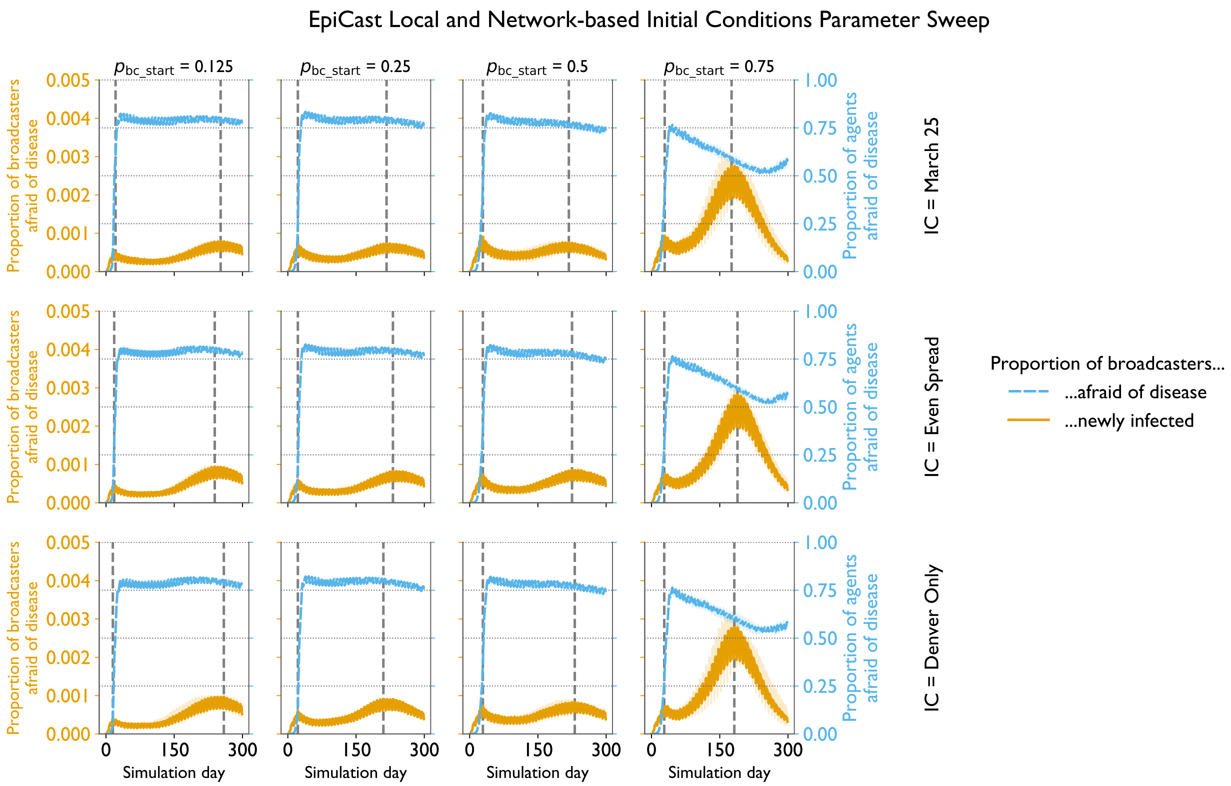

Supplementary Figure S3. **Epicast two-wave initial condition and broadcaster threshold sensitivity analysis:** New cases by day (with peaks indicated by dashed vertical line), for various initial conditions (IC) and thresholds for broadcasters to start spreading fear (p_{bc_start}) with both local and broadcaster-based fear spread. All lines represent the average of three replicates, with a 95% confidence interval shown in shading.

Variable	Description	Experiment					
		(a)	(b)	(c)	(d)	(e)	(f)
β	disease transmission rate	0.2	0.2	0.2	0.2	0.2	0.2
γ	disease recovery rate	0.5	0.5	0.5	0.5	0.5	0.5
p_s	probability of symptomatic infection			0.6 [1]	0.6	0.6	0.6
$p_a = 1 - p_s$	probability of asymptomatic infection			0.4 [1]	0.4	0.4	0.4
ι_a	relative infectivity of asymptomatic infection			0.75 [1]	0.75	0.75	0.75
δ	inverse of incubation period					0.5	0.5
β_f	fear transmission rate	1.1β [2]	1.1β	1.1β	1.1β	1.1β	1.1β
γ_f	baseline fear loss rate	0.05 [2]	0.05	0.05	0.05	0.05	0.05
α_f	fear loss contact rate	2.2β [2]	2.2β	2.2β	2.2β	2.2β	2.2β
ρ_f	relative fear susceptibility after symptomatic recovery	1	0 [2]	0	0	0	0
ι_f	relative infectivity when fearful	1 [1]	1	1	1	1	1
σ_f	relative susceptibility when fearful	0.25 [2]	0.25	0.25	0.35	0.25	0.35

Supplementary Table S1. Parameter values used in ODE model experiments: Results of experiments (a-e) are shown in the corresponding subfigures in Fig. 1, and the EpiCast scenarios to setups with (a) only hospitalization-based withdrawals, (b) the addition of symptomatic fearful withdrawals, and the addition of either pure-fear withdrawals from fear – without (c) or with (d) broadcaster-based fear spread – or reduced susceptibility when fearful – without (e) or with (f) broadcaster-based fear spread.

References

1. U.S. Centers for Disease Control and Prevention (CDC). https://archive.cdc.gov/www_cdc_gov/coronavirus/2019-ncov/hcp/planning-scenarios.html (2021).

2. Epstein, J. M., Hatna, E. & Crodelle, J. Triple contagion: a two-fears epidemic model. *J. Royal Soc. Interface* **18**, 20210186 (2021).

Variable	Description	Scenario (a)	(b)	(c)	(d)	(e)	(f)
β	disease transmission rate	0.2	0.2	0.2	0.2	0.2	0.2
γ	disease recovery rate	0.5	0.5	0.5	0.5	0.5	0.5
p_s	probability of symptomatic infection	0.6	0.6	0.6	0.6	0.6	0.6 [1]
$p_a = 1 - p_s$	probability of asymptomatic infection	0.4	0.4	0.4	0.4	0.4	0.4 [1]
ι_a	relative infectivity of asymptomatic infection	0.75	0.75	0.75	0.75	0.75	0.75 [1]
δ	inverse of incubation period	0.5	0.5	0.5	0.5	0.5	0.5
β_f	fear transmission rate	1.1β	1.1β	1.1β	1.1β	1.1β	1.1β [2]
γ_f	baseline fear loss rate	0.05	0.05	0.05	0.05	0.05	0.05 [2]
α_f	fear loss contact rate	2.2β	2.2β	2.2β	2.2β	2.2β	2.2β [2]
ρ_f	relative fear susceptibility after symptomatic recovery	0	0	0	0	0	0 [2]
ι_f	relative infectivity when fearful	1 [1]	1	1	1	1	1
σ_f	relative susceptibility when fearful	1	1	1	1	0.35	0.35
p_{sick}	probability of withdrawal when fearful with symptoms	0	1	1	1	1	1
p_{fear}	probability of pure-fear withdrawal when fearful	0	0	0.65	0.65	0	0
p_{bc}	probability of watching a given broadcaster	0	0	0	0.25	0	0.25
$p_{\text{bc\_start}}$	threshold of fearful workers for broadcasters to take position				0.5		0.5
$p_{\text{bc\_neutral}}$	relative rate of new cases for broadcasters to resume neutral position				$\frac{p_{\text{bc\_start}}}{2}$		$\frac{p_{\text{bc\_start}}}{2}$
$p_{\text{bc\_counter}}$	relative rate of new cases for broadcasters to counter fear spread				$\frac{p_{\text{bc\_start}}}{4}$		$\frac{p_{\text{bc\_start}}}{4}$

Supplementary Table S2. Parameter values used in EpiCast scenario experiments: Parameters represent scenarios with (a) only hospitalization-based withdrawals, (b) the addition of symptomatic fearful withdrawals, and the addition of either pure-fear withdrawals – without (c) or with (d) broadcaster-based fear spread – or reduced susceptibility when fearful – without (e) or with (f) broadcaster-based fear spread.
